# Altering alpha-frequency brain oscillations with rapid analog feedback-driven neurostimulation

**DOI:** 10.1371/journal.pone.0207781

**Published:** 2018-12-05

**Authors:** Alik S. Widge, Matthew Boggess, Alexander P. Rockhill, Andrew Mullen, Shivani Sheopory, Roman Loonis, Daniel K. Freeman, Earl K. Miller

**Affiliations:** 1 Department of Psychiatry, Massachusetts General Hospital and Harvard Medical School, Boston, Massachusetts, United States of America; 2 Picower Institute for Learning & Memory, Massachusetts Institute of Technology, Cambridge, Massachusetts, United States of America; 3 College of Engineering, Boston University, Boston, Massachusetts, United States of America; 4 The Charles Stark Draper Laboratory, Inc., Cambridge, Massachusetts, United States of America; Georgia State University, UNITED STATES

## Abstract

Oscillations of the brain’s local field potential (LFP) may coordinate neural ensembles and brain networks. It has been difficult to causally test this model or to translate its implications into treatments, because there are few reliable ways to alter LFP oscillations. We developed a closed-loop analog circuit to enhance brain oscillations by feeding them back into cortex through phase-locked transcranial electrical stimulation. We tested the system in a rhesus macaque with chronically implanted electrode arrays, targeting 8–15 Hz (alpha) oscillations. Ten seconds of stimulation increased alpha oscillatory power for up to 1 second after stimulation offset. In contrast, open-loop stimulation decreased alpha power. There was no effect in the neighboring 15–30 Hz (beta) LFP rhythm or on a neighboring array that did not participate in closed-loop feedback. Analog closed-loop neurostimulation might thus be a useful strategy for altering brain oscillations, both for basic research and the treatment of neuro-psychiatric disease.

## Introduction

Since the earliest days of electrophysiology, scientists have observed rhythmic oscillations of the brain’s electrical fields, generated by the synchronized firing of neural ensembles. More recently, those oscillations have been suggested as a mechanism for multi-scale neural communication [1–4]. Lower-frequency rhythms appear to implement long-range top-down control [5–10] while higher-frequency rhythms such as the gamma (~30–55 Hz) oscillation are more implicated in local and bottom-up processing [11–15]. These same oscillations have been suggested to become abnormal in neuro-psychiatric disease. The most well-known examples are pathological beta rhythms in Parkinson disease [16,17] and gamma abnormalities in schizophrenia [14]. A variety of oscillatory phenomena, particularly in the theta band, have been suggested as markers of depressive symptoms [18]. Altering or enhancing oscillations in the local field potential (LFP) might thus be a useful treatment approach for neuro-psychiatric disorders [19–23].

The challenge is that it is not straightforward to control the power or timing of brain oscillations. Primary sensory cortex entrains to rhythmic stimuli [24,25] but higher-order cortex often does not. Clinical technologies such as transcranial alternating current stimulation (tACS) [19,26–28] and deep brain stimulation (DBS) [29–32] apply rhythmic electrical stimulation, but this does not necessarily affect the corresponding cortical oscillation. The brain often responds by changing power in a different frequency band than the dominant frequency of the applied stimulation. DBS is delivered as high-frequency biphasic pulses to sub-cortical targets. High-frequency stimulation produces cortical oscillatory effects in lower frequency bands, although the exact band varies depending on the underlying disease. For example, common high-frequency DBS patterns change beta (15–30 Hz) oscillations [16,17] as well as beta-gamma coupling [33] recorded invasively in Parkinson disease patients. In psychiatric patients, by contrast, DBS effects on resting-state electroencephalographic (EEG) oscillations are inconsistent, with suggestions of both beta [34] and theta changes [35]. tACS, in contrast, more directly targets cortex. tACS seems most effective at increasing oscillatory power when it is well-matched to the target’s endogenous frequency profile [36–38]. For instance, frequency-matched tACS has enhanced cognition [37] and can augment brain network connectivity in slow oscillatory regimes [39]. The same effect may decrease oscillatory power in cases where the stimulation and a physiologic oscillation naturally oppose each other [40].

One logical approach to altering oscillatory power would be to time-lock stimulation to the phase of an ongoing rhythm. Stimulating at oscillatory peaks should constructively interfere with and enhance an ongoing oscillation, whereas stimulating mainly at oscillatory troughs should destructively interfere. A key difficulty, however, has been in accurately estimating oscillatory phase in real-time. By the time a signal is digitized and its phase computed through Fourier-transform-based algorithms, the target phase has long since passed by [41]. In recent animal studies that sought to use the phase-locking approach, stimulation was 90–180° away from the target phase [42,43]. A faster, but more technically challenging approach, would be to deliver phase-locked stimulation through analog circuitry. Analog feedback circuits were once indispensable neuroscience tools [44], but with the advent of high-speed analog-to-digital converters, most modern applications switched to digital processing. Here, we sought preliminary proof-of-concept that an analog feedback system could modify cortical brain oscillations.

## Methods

### Alpha inverting filter

We targeted the alpha (8–15 Hz) oscillation, which has been implicated in top-down cortical processing [6,45,46] and which has a wide enough bandwidth for analog filtering to be practical without excessive phase distortion. Alpha is also a target of interest because it has been shown to change in response to transcranial stimulation in humans [26,47,48]. Like all named EEG frequency bands, the precise boundaries of alpha are variable between investigators. We chose a definition of alpha consistent with our past research [22,49].

**Fig 1:**
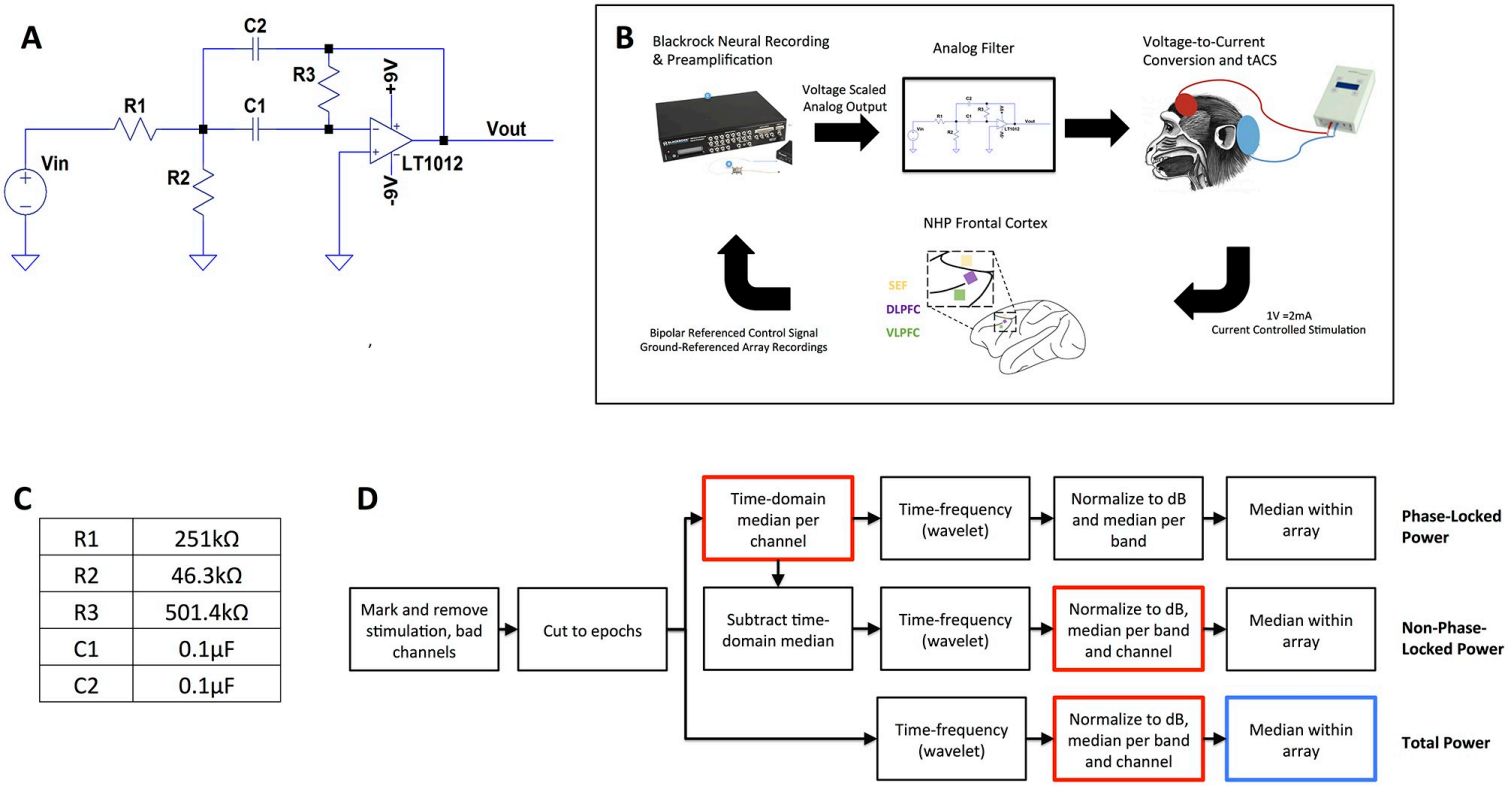
*In vivo* experiment design. (**A**), circuit diagram of bandpass filter for oscillation-related feedback. The op-amp’s output could be coupled back to its input for direct voltage feedback, or to an external stimulator for isolated and/or optical stimulation. V_in_ and V_out_ are the input (LFP) and output (stimulation control) of the feedback system, respectively. (**B**), block diagram of *in vivo* testing. Signals were pre-amplified through a Blackrock Cerebus neural recording system, then fed through the analog filter and back to the brain through a voltage-controlled constant-current transcranial stimulation system. The inset shows the position of the supplemental eye field (SEF), dorsolateral prefrontal cortex (dlPFC), and ventrolateral prefrontal cortex (vlPFC) microelectrode recording arrays relative to major sulci. The macaque diagram shows the approximate location of tACS electrodes, with a smaller stimulating electrode placed on the scalp over the recording implants and a larger return electrode on the back of the head. In this paper, the SEF array was Array 1 and the dlPFC array was Array 2. (**C**), resistor and capacitor values used to realize the circuit for *in vivo* testing. (**D**), flow chart of analysis. After removal of stimulation artifact periods and bad channels, data passed through three separate streams to extract the total, phase-locked, and non-phase-locked power within the alpha, beta, and high-gamma frequency bands. Two sets of bootstrap/permutation testing were performed. The first tests for differences between conditions (Open, Brain, Closed) and whether each condition differs from zero effect. Red boxes within the flowchart highlight the permutation point, where labels are randomly shuffled. The second, performed only on the total power, tests for differences between Array 1 and Array 2.

We designed an analog circuit to extract and invert the sign of neural oscillations (Fig 1A) with an active inverting band-pass filter. The pass band was set slightly wider than alpha, at 8–16 Hz (center frequency 12 Hz). Traditionally, cascaded second-order Sallen-Key or multiple feedback (MFB, Eq 1) topologies are used to achieve a narrow filter bandwidth. Cascading multiple filters together allows for sharp power roll-off at the passband boundaries. However, cascades cause rippling at the boundaries of the filter and increase the phase distortion. We sought to minimize the phase shift at the center frequency, eliminating the cascaded topology and limiting us to a single-stage active band-pass filter. This shallow roll-off led to the wider passband but reasonably met our defined range of alpha. For a single-stage filter, the MFB topology was preferable to Sallen-Key for two reasons. First, even though 1 Hz wider than the traditional alpha definition, our filter bandwidth of 8 Hz was overall quite small. Because bandwidth and quality factor are inversely related, the circuit had high quality factor. At high quality factors, Sallen-Key topologies have higher ripple at the passband boundaries compared to MFB; we therefore preferred MFB for this specific application. Finally, the MFB topology is naturally inverting, eliminating the need for an inverter in our analog processing chain.

$$A(s)=\frac{-\frac{R_{2}R_{3}}{R_{1}{+R}_{3}}C\omega_{m}∙s}{1+\frac{{2R}_{1}R_{3}}{R_{1}{+R}_{3}}C\omega_{m}∙s+\frac{{R_{1}R}_{2}R_{3}}{R_{1}{+R}_{3}}C^{2}∙{\omega_{m}}^{2}∙s^{2}}$$ (1)

Eq 1 The transfer function of an MFB second-order bandpass filter

To realize this filter design, we specified the Linear Technology LT1012 op-amp, designed for low-frequency active filter applications. This had a low intrinsic noise, 0.5 μV peak-to-peak over 0.1–10 Hz, and a typical input bias current of 5 pA. LFP signal levels were expected to be on the order of 500 μV, providing significant signal to noise ratio. Low bias current is valuable since we would like to minimize the applied current when stimulation is not desired. We verified the output of the filtering and feedback circuit by simulating its response to pre-recorded LFP data in LT-SPICE (Linear Technology, Milpitas, CA). To further verify that we achieved the desired phase response for alpha signals, we also simulated the response to pink noise that had been pre-filtered into the alpha band (MATLAB "filtfilt" function with a second-order Butterworth bandpass filter, passband as per the realized circuit).

### Closed-loop neural system

We coupled the feedback circuit to an *in vivo* neural recording chain (Fig 1B). Because the designed circuit lacked a pre-amplifier, we connected it to the output of a CerePlex amplifying head stage (Blackrock Microsystems, Salt Lake City, UT). The CerePlex output is digital, and we re-converted this to the analog domain within a Cerebus Neural Signal Processor (NSP) and passed the signal out a front-panel port of that NSP. The feedback circuit’s voltage output then controlled the current delivered by a DC-Stimulator Plus (NeuroConn GmbH, Ilmenau, Germany), at a ratio of 2 mA per applied volt. The DC-Stimulator Plus limits voltage applied to each electrode to prevent electrolytic or other detrimental effects. This created a closed feedback loop where current could be applied to a living brain in proportion to its specific oscillatory frequency and phase. The pre-amplifier and head stage added delay to the processing chain. The total phase delay of the system was 372° at the center frequency of 12 Hz and was exactly 360° at 11.6 Hz. This represents a 185° shift from the amplification/inversion circuit plus a total delay of 175° (42 ms) from the digital components. Due to the integrated/proprietary nature of the Blackrock system, we are not able to further break that delay into the independent contributions of the head stage, pre-amplifier, or internal processing of the neural signal processor. The effect of these summed delays was to create a positive feedback loop that should amplify alpha oscillations by applying a stimulation that is nearly in-phase (albeit shifted by one cycle) with the endogenous generator. The approach could be generalized to other frequencies by adjusting the filters and the overall loop delay. For instance, theta oscillations are commonly implicated in cognitive control; they could be augmented by adjusting the resistors and capacitors to yield a passband of approximately 5–8 Hz with a phase shift of approximately 270° at 6 Hz (because the 42 ms digital delay is equivalent to 90° phase shift). If necessary, this could be realized by combining the bandpass stage with an all-pass filter designed to provide additional phase delay. Of note, the analog circuit itself was originally designed to suppress or cancel alpha oscillations through direct negative feedback. Unfortunately, the circuit as designed and realized could not operate without amplification. The digital amplification necessarily added further phase delay, and we thus shifted to a positive feedback experiment. In future designs, it should be possible to incorporate amplification directly into the circuit, without digitization. This would eliminate that additional phase delay and may permit feedback that cancels oscillations, e.g. the pathological beta oscillation commonly seen in Parkinson disease.

### In vivo testing: Animal details

We tested feedback alteration of neural oscillations by stimulating in closed loop to the prefrontal cortex (PFC) of a male rhesus macaque approximately 10 years of age. The animal was originally received from a macaque breeding colony in California. All experimental procedures were approved by the Massachusetts Institute of Technology Committee on Animal Care, which provides ethical review of all animal research at our institution. Three 8x8 channel Blackrock Cereport arrays with 1mm long electrodes were placed within dorosolateral prefrontal cortex (dlPFC), ventrolateral prefrontal cortex (vlPFC), and the frontal and supplementary eye-fields regions (FEF, SEF) (Fig 1B, inset). Note that the structure of this 8x8 array (derived from a 10x10 grid with corners removed) yields 96 electrodes in total. Electrodes were separated by 400 μm. vlPFC and dlPFC were defined by anatomical landmarks following a large craniotomy. 3D MRI brain reconstructions and plastic models were used to guide the surgical implants of the array. The dlPFC array was positioned 12–15 mm anterior to the genu of the arcuate and 1 mm dorsal to the principal sulcus. The craniotomy was closed with a bone flap after array and pedestal placement.

To monitor the onset of post-operative morbidity, the macaque was monitored every 15 minutes after the procedure until he demonstrated orienting competently. He was monitored every 30 minutes until motor balance was restored. At that point, he was returned to his home cage. Food/fluid intake was monitored twice daily and returned to pre-surgical baseline. No post-operative infection was observed. To reduce post-operative distress, we administered buprenorphine (0.02 mg/kg) at first surgical incision and then again every 12 hours for 48 hours post-operation. Cefazolin (25 mg/kg) was given pre-operatively as an antibiotic to reduce infection. We supplemented buprenorphine analgesia with carprofen (3 mg/kg) for 48 hours post-procedure. Before surgery and after recovery, the macaque was pair-housed with a male conspecific in a larger room containing multiple NHPs. The macaque was provided ad libitum access to water. Facility veterinary staff provided a daily ration of chow biscuits optimized to maintain healthy weight and nutritional status. Veterinary staff provided, on a daily basis, several forms of environmental enrichment (such as puzzles & toys) specifically designed for nonhuman primates. Treats, including fresh fruit, were also available as part of the enrichment program. The housing room contained a television that aired programs featuring monkeys, chimpanzees, and other animals. The macaque housing room was on a 12-hour light/dark schedule. While participating in this study, the macaque had a quarterly health examination that showed no deficits. After participation in this study, the macaque remained implanted and continued on to perform other studies in the same laboratory [50].

### In vivo testing protocol

We selected a pair of electrodes on the array in the supplemental eye field (SEF), which we refer to as "Array 1", and converted them to a single bipolar-referenced channel. This channel, as pre-amplified by the Cerebus system, formed the basis for closed-loop feedback. We refer to it as the "controller" channel. Importantly, because this bipolar channel was formed from two electrodes of very similar impedance, it offered common-mode rejection that cancelled the stimulation artifact and made continuous closed-loop feedback possible. All 96 electrode channels on each array, including the controller channel, were digitized at 1000 Hz and separately stored. These recordings were referenced to the animal’s titanium head post. No electrodes on any of the microelectrode arrays were used for stimulation.

The macaque underwent four days of testing with the same controller channel. On each testing day, we fixed the animal’s head in place by an implanted titanium headpost and accessed the arrays through chronic head-mounted connectors. We then placed rubber stimulation electrodes according to surface landmarks on the right frontal scalp over the implanted arrays (3 x 3 cm) and on the back of the animal’s neck (5x7 cm) and connected these to the DC-Stimulator output. Electrical contact between the rubber electrodes and skin was established with Grass EC2 conductive cream. The wires of each stimulation electrode were directed posteriorly towards the headpost and the animal’s occiput. The DC-Stimulator voltage-to-current gain is fixed, but the NSP can convert a digitized channel to analog with variable gain. If this variable gain is too high, the closed-loop filter can exceed its phase/gain margins and create a positive feedback loop. On each testing day, we titrated the gain to a level that did not cause feedback, then left the animal un-stimulated for at least 10 minutes before beginning trials. This resulted in absolute output voltages under 500 mV, which by the DC-Stimulator’s 1V/2mA gain meant that stimulation amplitude varied continuously within the range of ±1 mA.

We tested three types of stimulation: closed-loop stimulation using our filter (Closed), open-loop constant sinusoidal stimulation at 11.5 Hz (Open), and open-loop stimulation using an 8–16 Hz filtered neural waveform recorded from the controller channel on a previous day (Brain). All were applied such that the stimulation waveform remained within the stimulator compliance window without clipping. We applied each stimulation type in trials of 10 seconds followed by 40-second rest intervals. We chose this fairly short-duration intervention to avoid any concern for seizure or other adverse neurologic effect of a previously-unknown intervention. Stimulation onset and offset were manually controlled by a human experimenter (AM or RL) using a stopwatch. Stimulations occurred as blocks of 10 trials followed by 120 seconds of rest between conditions. The order of condition blocks was determined by a random number generator, and each condition was tested twice on each testing day. Two exceptions were that the Brain condition was not tested on the first day (because no recording was yet available) and that each condition was tested four times on the final day. The four days of recording resulted in 100 trials of Open and Closed stimulation and 80 trials of Brain.

### In vitro testing

To verify that any observed stimulation effects were not due to changes in electrode properties, we tested the same stimulation paradigm in a saline beaker containing no endogenous electrical field generators. We placed 250 mL of 0.9% sodium chloride solution into a glass beaker and immersed a 96-channel silicon microelectrode implant (Blackrock Cereport array, identical to those used *in vivo*) in the solution. The array had not previously been implanted in an animal. We placed the anode and cathode of the DC-Stimulator Plus on the other side of the beaker from the microelectrode array.

We selected a pair of adjacent electrodes on the array and converted them to a single bipolar-referenced channel. This was analogous to the bipolar-referenced channel used to measure local field potentials from PFC during *in vivo* testing. All electrode channels were digitized at 1000 Hz. The recordings were referenced to a metal beaker stand that the glass beaker was sitting on.

The beaker received three forms of stimulation: closed-loop stimulation using our filter, open-loop constant sinusoidal stimulation at 11.5 Hz, and open-loop stimulation using an 8–16 Hz filtered neural waveform recorded from the macaque in the *in vivo* experiments. For each stimulation paradigm, we applied trials of 10 seconds of stimulation followed by 40 seconds of rest, again determined by the use of a stopwatch. Stimulations occurred as blocks of 30 trials followed by several minutes of rest.

### Analysis

The raw Blackrock data files were imported into the MNE-Python suite [51], after which analysis was conducted through a combination of core MNE-Python functionality and custom Python scripts. The overall analytic chain is shown in Fig 1D. The raw data were epoched from -5 to +5 seconds surrounding both the stimulation onsets and offsets. Those onset and offset events were marked manually based on visual inspection for the point at which the amplifier had de-saturated. Note that this saturation did not occur on the bipolar controller channel. Saturation was limited to the remainder of the array channels that were recorded in single-ended mode. No artifact rejection or correction was performed post-recording. We did reject trials where stimulation was not faithfully or correctly applied, i.e. any trials where the elapsed time between onset and offset exceeded 11 seconds *in vivo* and 20 seconds *in vitro* as the amplifier took longer to desaturate *in vitro*. We removed the controller channel and its reference from the dataset before analysis. We also removed, in both *in vivo* and *in vitro* recordings, channels known to be non-functioning (as determined either by unusually high impedance or flat recording traces).

The epoched data were transformed into a time-frequency representation using Morlet wavelet convolution. Each individual trial was convolved with a set of complex Morlet wavelets that increased linearly in both frequency (from 5 to 30 Hz in steps of 1 Hz) and number of wavelet cycles (from 5 to 30 cycles in steps of 1 cycle). Power was then extracted as the squared magnitude of the result of the convolution. This was done separately for both the stimulation onset and offset epochs. The last 5 seconds of the stimulation onset epochs and the first 5 seconds of the stimulation offset epochs (i.e, the period of active stimulation) were then dropped. On channels other than the bipolar controlling pair, these time periods were composed mainly of artifact. The remaining epochs, comprising 5 seconds before stimulation and 5 seconds after offset, were concatenated. This left us with 10-second epochs consisting of the 5 seconds prior to and after stimulation on each trial.

We normalized these power data with a slightly modified version of the procedure suggested by Grandchamp & Delorme [52]. For each wavelet frequency, each epoch was normalized by the median of the epoch. Next, at each timepoint, we calculated the median value across all epochs for each frequency. Finally, we calculated the grand median value of a baseline period of -5 to -1 seconds prior to stimulation. We then normalized the power data at all timepoints and frequencies by dividing by the respective baseline median. Finally, we transformed to decibels. Alpha, beta, and high gamma band power were extracted by averaging across all frequencies between 8–15 Hz, 15–30 Hz, and 80–200 Hz respectively. We chose these band definitions to remain consistent with analyses in our prior work and so that the beta band would be largely outside the feedback filter’s passband. The beta band thus would not be expected to change, even if alpha power changed from one or more of our stimulation protocols. The high gamma power (HGP), on the other hand, might change if our stimulation altered overall neuronal excitability. HGP has been suggested as an LFP correlate of regional spiking activity [53]. Finally, we averaged band activity in a window from 0.5 to 1 second after the stimulation offset to summarize post-stimulation effects, as we expected the effects of this relatively brief stimulation to be similarly brief. This process was done separately for each condition. All epochs in the same condition were pooled together from all days of recording.

To test whether each stimulation condition changed band-specific power relative to baseline, we averaged each band’s power across all electrodes on the array that controlled stimulation (Array 1), with the exception of the controller channel itself. We then used a bootstrap test for post-stimulation-offset power modulations. The non-parametric permutation approach protects against the frequently non-Gaussian distribution of electrophysiologic data, and it may have more statistical power for our relatively small analysis window. Bootstrap samples were generated by resampling the raw time-frequency power trials with replacement 5000 times and calculating the average post-stimulation band power. This yielded a confidence distribution for the mean effect of each stimulation type on these bands. The fractional mass of that confidence distribution above zero yielded the p-value. To test for differences between stimulation conditions, we used a permutation test. For each stimulation condition pair, we permuted the condition labels on the raw-time frequency power 5,000 times and calculated the difference in post-stimulation band power. This yielded a null distribution for the difference between specific conditions. For comparisons with the Brain condition, we first randomly subsampled the Open and Closed conditions to 80 trials before each permutation, to equalize the sample sizes. The p-values were calculated as the fraction of values in the permutation null distribution whose absolute value was greater than or equal to the absolute value of the non-resampled post-stimulation power difference between the two conditions. We report both uncorrected and false-discovery-rate (FDR) [54] corrected p-values, where we performed FDR correction for multiple pairwise tests of conditions within each frequency band (i.e., across 3 comparisons at a time).

We recorded signals from multiple arrays, spaced several millimeters apart, during the stimulation experiment. The local field potential varies in power and frequency content across millimeters of cortical tissue, meaning that Closed stimulation should only be well-matched to the alpha rhythms of the array containing the controller electrodes (Array 1). We therefore hypothesized that post-stimulation-offset changes would be different between arrays. To test this, we compared the array containing the controlling pair to the array implanted over dlPFC (Array 2), again using a permutation test. We generated 5,000 permutations of array assignments by randomly shuffling the array membership of each electrode, then took the difference of the average post-stimulation power in each band between the two arrays. This corresponds to the blue-framed step in Fig 1D. The permutation p-value was computed as the fraction of values in the permutation null distribution whose absolute value was greater than or equal to the absolute value of the corresponding difference in the non-permuted data. We report both uncorrected and FDR corrected p-values, pooled by band, in this analysis also.

Finally, we considered the question of whether any resulting LFP changes were phase locked to the stimulation. In past reports of oscillatory enhancement with pulsatile transcranial magnetic stimulation, a beta oscillation both increased its power and became phase-locked to the stimulation [55]. To test for this possibility, we repeated the above analysis chain specifically for phase-locked (PL) and non-phase-locked (NPL) power, following an approach outlined in [56]. For PL activity, rather than time-frequency transforming individual trials, we first took the median voltage at each timepoint for all trials within a condition (effectively creating an evoked potential). We then time-frequency transformed that phase-locked activity. For NPL, we subtracted that evoked potential from each individual trial before time-frequency transformation. These analysis chains are also schematically depicted in Fig 1D.

All analysis code will be available after publication at https://github.com/mghneurotherapeutics/GalileoTACS and the corresponding data at https://osf.io/f6nqx/?view_only=db8001c6853a45a6b682b6f56e128a97.

## Results

**Fig 2:**
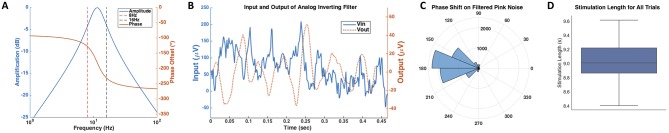
Characterization of analog feedback system for neural oscillation modification. (**A**), Bode diagram of filter response as simulated in LT-SPICE, showing rapid roll-off and linearized phase behavior in the least-attenuated portion of the passband. (**B**), filter performance on a representative sample of pre-recorded data (V_in_ = input, V_out_ = output), demonstrating extraction of alpha (8–16 Hz) activity and sign inversion. This trace also provides an example of a waveform that would be applied as the stimulation in the Closed and Brain conditions. (**C**), phase response of filter on a signal (pink noise filtered to the alpha band) with predominant power within the filter passband. The histogram shows the difference between the phase of the signal provided at V_in_ and the phase at V_out_, as computed via Hilbert transform on both signals. The phase shift of the filter is, as desired, predominantly at or near 180°. (**D**), timing of the stimulation periods for the *in vivo* experiment. The mean was 8.97 seconds, versus a target of 10 seconds, with all stimulation periods between 7.74 and 10.74 seconds in length.

The filter as designed exhibited a sharp passband, and the phase shift within that passband was nearly linear (Fig 2A). We then simulated the filter’s output on a pre-recorded sample of LFP data. As expected, the output was an inverted version of the low-frequency (alpha) peaks, i.e. a roughly 180° phase shift (Fig 2B and 2C). In the animal testing, this inverted signal underwent a further phase shift from the pre-amplification and digitization, putting the peak stimulation intensity back into phase with the ongoing brain rhythms. During that testing, we aimed for 10 seconds of tACS and achieved slightly under this, with a mean stimulation period of 8.97 seconds (range, 7.74 to 10.74 seconds, Fig 2D).

**Fig 3:**
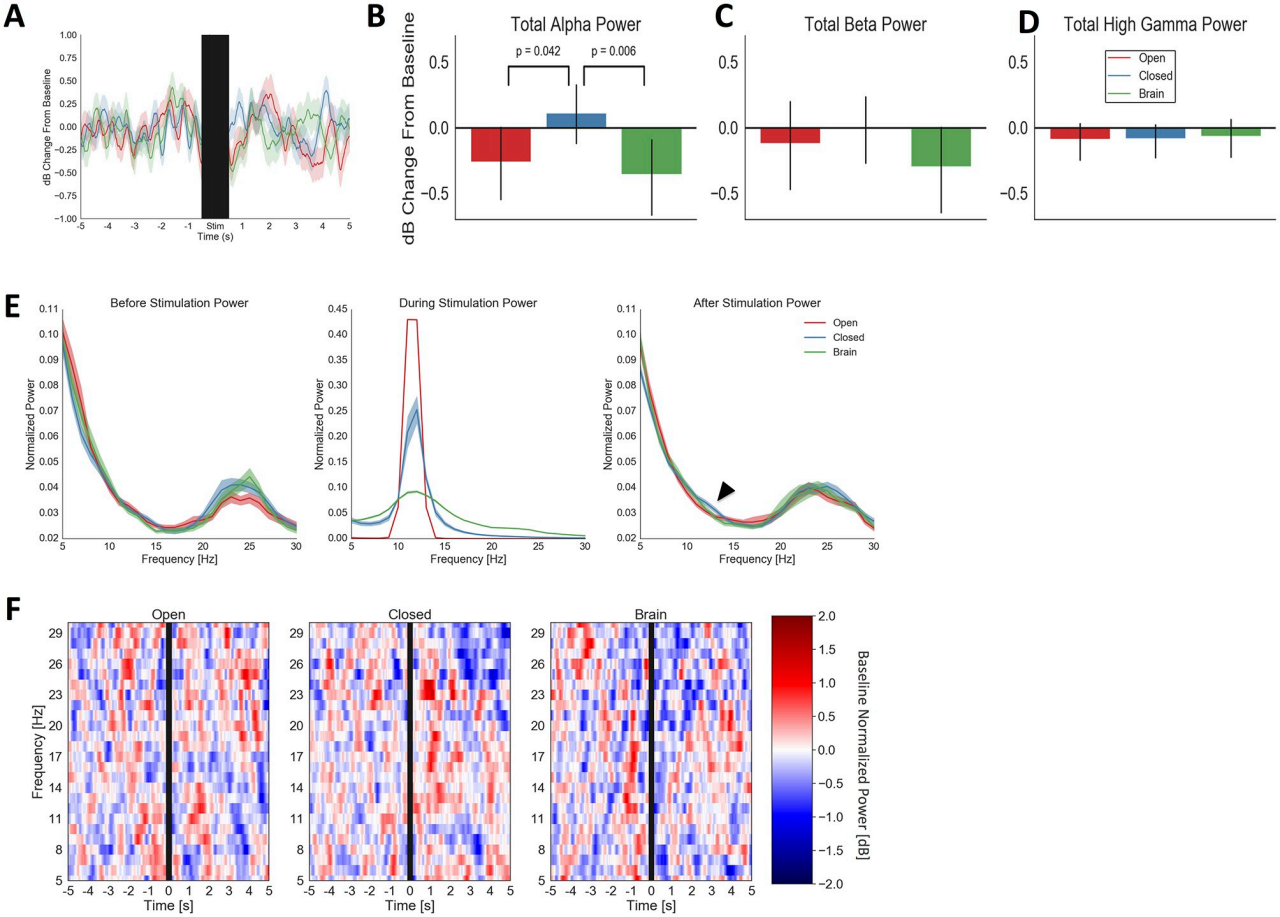
Physiologic response to analog feedback (Closed) and open-loop stimulation (Open, Brain) on Array 1. (**A**), average pre/post stimulation traces with confidence bounds (1 standard deviation) computed by bootstrap resampling. The stimulation period (10 seconds ± 0.5 seconds to mask residual stimulation artifact) is indicated by a black bar. During the period immediately after stimulation offset, Closed-loop stimulation slightly increased alpha power over baseline, while open-loop controls decreased alpha power. (**B**), mean alpha power from 0.5 to 1 second after stimulation offset, further demonstrating Closed-loop alpha enhancement and Open-loop/Brain-based suppression. Closed differs from Open (p = 0.042 uncorrected; p = 0.126 FDR) and Brain (p = 0.006 uncorrected; p = 0.017 FDR) conditions on permutation testing, but not from its baseline in this analysis (p = 0.379 uncorrected, p = 0.491 FDR corrected). Error bars represent full 95% confidence interval from bootstrap resampling. (**C**), mean beta-band power during same period as (B). No condition has a significant change from its baseline or from another condition (all p > 0.07 uncorrected), suggesting that alpha-frequency stimulation preferentially affected alpha-band activity. (**D**), mean high-gamma-band power during same period as (B). No condition significantly differs from its baseline (all p > 0.210 uncorrected, 0.350 FDR corrected) or from another condition (all p > 0.571 both uncorrected and FDR corrected), suggesting that alpha-frequency stimulation did not directly affect spiking on Array 1. (**E**), normalized spectral densities of LFP from 1 to 0.5 seconds before stimulation (Before), the stimulation period (During), and 0.5 to 1 second after stimulation (After), for all three tested conditions. The conditions do not differ in alpha power before stimulation, but the subtle alpha enhancement from the Closed condition (the effect plotted in panel B) is visible in the rightmost panel. We have further highlighted this with an arrowhead. The blur about each line represents standard error of the mean for spectral estimate at each frequency, as calculated from 5,000 bootstrap samples with replacement. (**F**), time-frequency representations of Array 1 power, with the same data and time windows as in panel A. For clarity, only beta and below are shown. The changes plotted in the previous panels are not narrow-band but instead appear to occur throughout the alpha band.

All three brain stimulation regimes changed alpha power on Array 1 (Fig 3A). Immediately after stimulation offset, for up to 1 second, stimulation that was not phase-locked to the ongoing alpha oscillation (the Brain and Open conditions) decreased alpha power from baseline. Closed-loop stimulation, by contrast, increased alpha power. On permutation testing, alpha power from 0.5 to 1 second after Closed stimulation was significantly greater (Fig 3B) than after Brain (p = 0.006 uncorrected; p = 0.017 FDR) and Open (p = 0.042 uncorrected; p = 0.126 FDR) stimulation. The effects were frequency-specific; no stimulation modality differed from another in beta-band or high gamma power (Fig 3C and 3D; all p > 0.160 uncorrected). This is also seen when examining spectral densities before and after stimulation (Fig 3E). The effects support the hypothesis that in-phase Closed-loop stimulation would enhance the alpha rhythm, while control stimulations would interfere with and reduce alpha. Stimulation effects were relatively short-lived; we found no significant differences in any analysis when testing a larger window from 0.5 to 1.5 seconds after stimulation offset. Effects were not, however, sensitive to electrode selection within the array. We repeated the analysis using a "spotlight" around the controlling pair, namely all electrodes with a Manhattan distance ≤ 2 from the controlling pair. This did not change the significance pattern relative to testing with the whole array. Power changes in response to the stimulation conditions were relatively broad-band, with no evidence of specific peaks near the stimulation frequency (Fig 3F).

**Fig 4:**
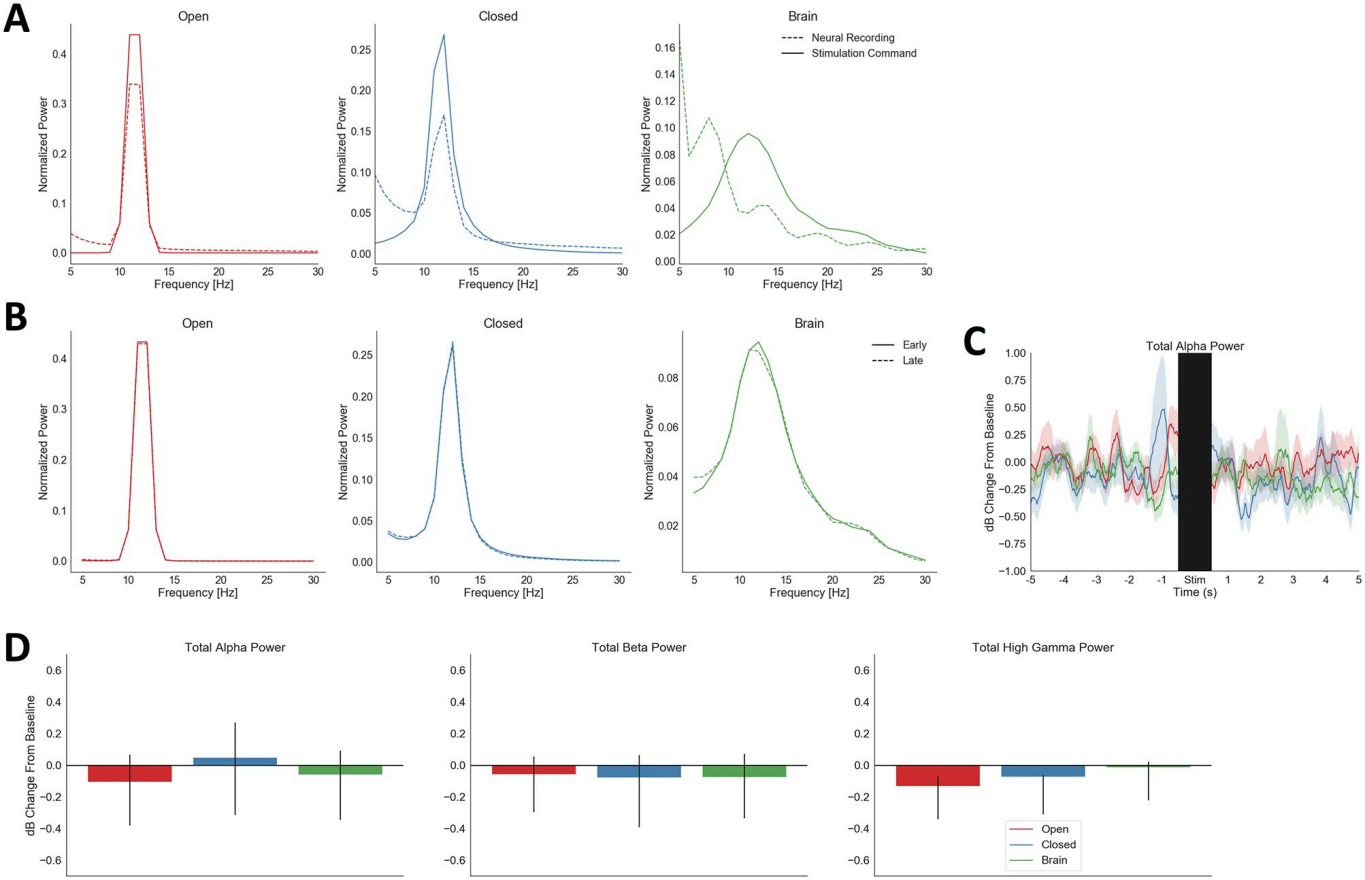
Verifications of correct system operation *in vivo* and *in vitro*. (**A**), normalized spectra of neural recordings from the bipolar-referenced channel pair used for stimulator control, during the stimulation period, in all three tested conditions. These are plotted as dashed lines; the command voltage to the neurostimulator (the output of the filtering/feedback circuit) is overlaid as a solid line. In all three conditions, the neural recording contains substantial frequency content outside the filter passband and does not precisely match the command spectrum within the passband, evidence that the recorded signal was not simply a copy of the stimulator artifact. (**B**), mean normalized spectra of neural recordings across the entire Array 1, excluding the controlling pair, in all three tested conditions, for the first and the last 5 s of stimulation. Spectra are not appreciably different between the two halves of stimulation, suggesting a lack of wash-in effects. (**C**), average pre/post stimulation traces from *in vitro* saline testing, with plotting conventions as in Fig 3. The stimulation and amplifier settling period is again indicated by a black bar. Unlike the *in vivo* results, confidence intervals of all traces overlap after the stimulation period. (**D**), mean alpha, beta, and high gamma power from 0.5 to 1 second after stimulation offset in the *in vitro* saline test, with plotting conventions as in Fig 3. No condition differs from its pre-stimulation baseline (all p > 0.162 uncorrected, 0.485 FDR) or from another condition (all p > 0.109 uncorrected, 0.328 FDR). Although the confidence interval for Open-loop stimulation does exclude zero in the high gamma analysis, it does not do so sufficiently to reach the pre-determined significance threshold.

We verified that the controlling pair was able to record neural signals even in the presence of stimulator artifact, by comparing the spectrum of the stimulator command to that of neural data simultaneously recorded on the controlling pair. In Open, Brain, and Closed conditions, these spectrograms had large areas of non-overlap (Fig 4A). The spectral properties of the stimulation did not change from the early (first 5 seconds) to late (final 5 seconds) stimulation period (Fig 4B). In contrast to the *in vivo* testing, the *in vitro* testing showed no significant changes in alpha, beta, or high gamma power within a condition or between conditions (Fig 4C and 4D). The only observed effect in the *in vitro* conditions was a longer settling time of the amplifier; the median period from stimulation start to amplifier settling was 16.5 seconds. This may represent an increased susceptibility of the bath preparation to polarization effects.

**Fig 5:**
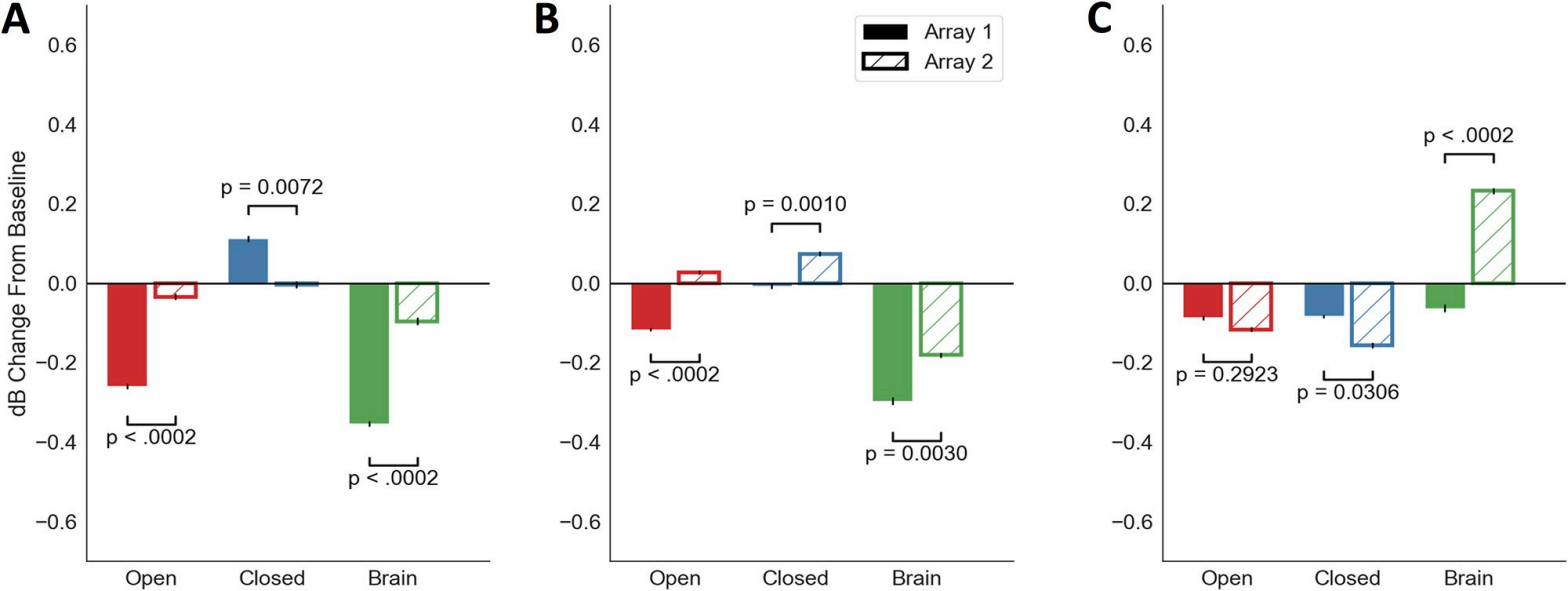
The response to stimulation, especially closed-loop, differs between the array used for closed-loop control (Array 1) and a more distant set of electrodes (Array 2). (**A**), alpha power changes between 0.5 and 1 s after stimulation offset. The increase in alpha with Closed-loop stimulation was specific to Array 1 (p = 0.007 uncorrected, p = 0.011 FDR, permutation test). Changes from Open-loop and Brain-based stimulation were non-specific, with both arrays decreasing from baseline. Array 1 effects were still stronger (both p < 0.0002 uncorrected, p = 0.0003 FDR, permutation test). Error bars again represent 95% confidence interval inferred from bootstrap resampling. They are narrower than in Fig 3 because the unit of permutation is the array, and arrays had less internal variability than did stimulation conditions. (**B**), beta power changes between 0.5 and 1 s after stimulation offset. Array 2 shows non-specific changes to Closed-loop stimulation not present on Array 1 (p < 0.003 uncorrected and p < 0.031 FDR for difference between arrays in all conditions, permutation test). (**C**), high gamma power changes between 0.5 and 1 s after stimulation offset. Effects are again non-specific, with Array 2 showing a decrease from Closed-loop stimulation and increase from Brain pattern stimulation relative to Array 1.

In the same 0.5–1 seconds immediately after the stimulation period, stimulation of all types affected Array 1 (the controlling array) alpha band power more strongly than the neighboring Array 2 (Fig 5A; all p < 0.007 uncorrected and p < 0.011 FDR for permutation test of difference between array means). For Closed stimulation in particular, the alpha power increase was specific to Array 1, changing by 0.1 dB on Array 1 and only -0.007 dB on Array 2 (p = 0.007 uncorrected, p = 0.011 FDR). Consistent with the effects of Fig 3A, this was the only alpha power increase we observed, across groups and arrays. Array 2 instead showed a beta power increase to Closed-loop stimulation, a further non-specific effect (Fig 5B; 0.005 dB beta change in Array 1 vs. 0.097 dB in Array 2, p = 0.001 uncorrected and p = 0.003 FDR). As expected, Open-loop and Brain-based stimulation were less specific. They showed Array 2 changes in the same direction as their Array 1 effect, albeit of smaller magnitude (Fig 5; -0.26 dB vs. -0.028 dB for Open-loop alpha, p < 0.0002 uncorrected, p = 0.0003 FDR; -0.351 dB vs. -0.092 dB for Brain-based alpha, p < 0.0002 uncorrected, p = 0.0003 FDR). Array 2 changes were generally smaller than Array 1 changes in the alpha and beta bands. In high gamma, the pattern reversed, with larger Array 2 changes in the Closed and Brain conditions (p = 0.003 uncorrected, p = 0.031 FDR, p < 0.0002 uncorrected, p = 0.0003 FDR). The pattern of effects did not change when we expanded the analysis window to 0.5–1.5 s post-stimulation-offset.

**Fig 6:**
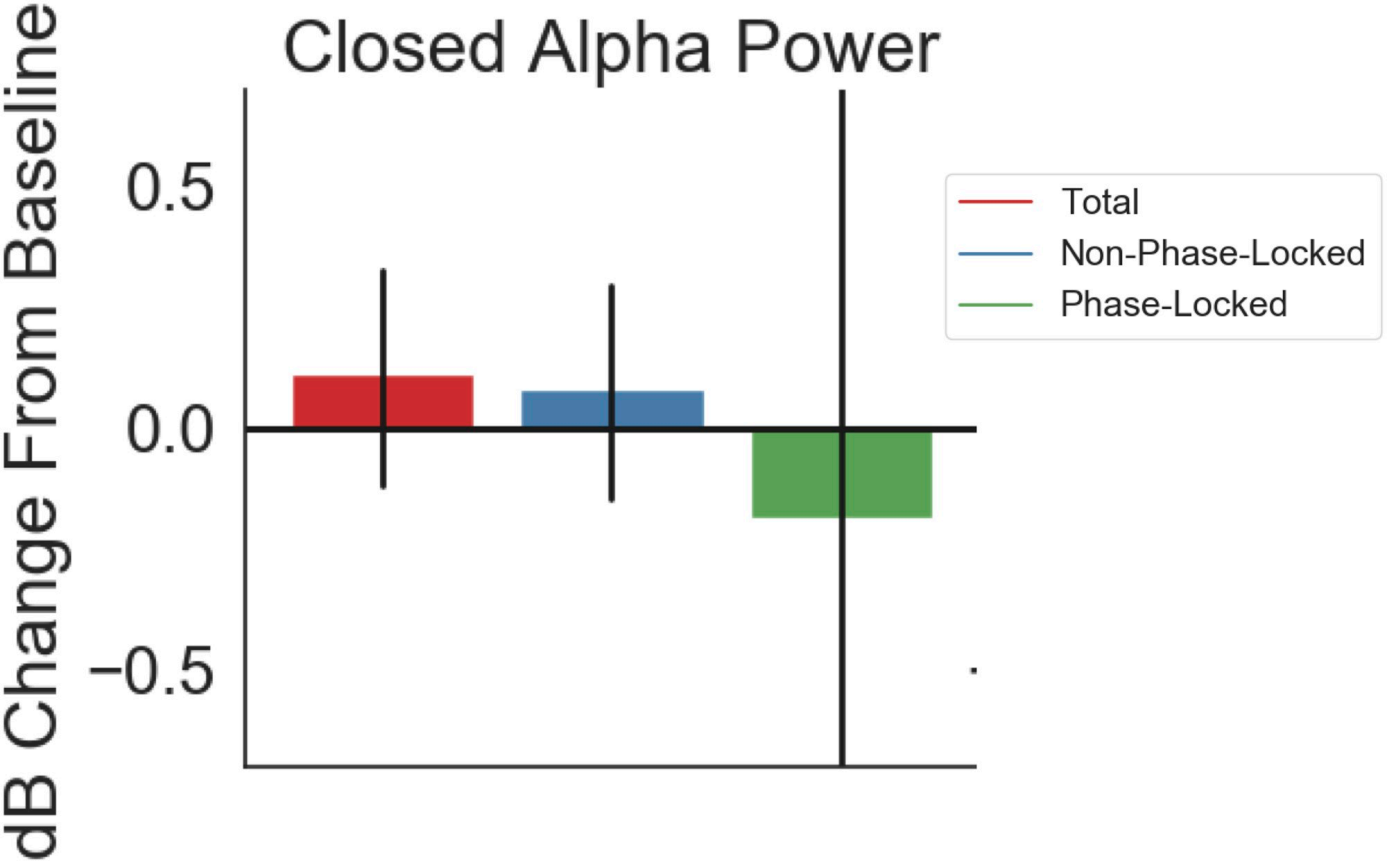
Power changes from closed-loop stimulation were not phase-locked to stimulation. Shown is the response to closed-loop stimulation in the alpha band, i.e. the same data analyzed in Fig 3A, now represented as change in total power, phase-locked power, or non-phase-locked (total–phase-locked) power. 74% of the total power change (0.113 dB) is in the non-phase-locked power (0.084 dB). Phase-locked change has a wide confidence interval suggesting essentially zero effect.

The observed alpha enhancement on Array 1 was not driven by entrainment of the endogenous alpha rhythm to the applied stimulation. When we broke the results of Fig 3B down by PL, NPL, and total power, NPL and total power changed in the same direction and by similar amounts: 0.113 dB for total power and 0.084 dB NPL (Fig 6). PL power, on the other hand, showed a decrease relative to baseline (-0.186 dB), albeit with a very wide confidence interval suggesting no true change.

## Discussion

Consistent with our initial hypothesis, closed-loop analog feedback stimulation enhanced brain oscillations in the target 8–15 Hz frequency band. Those changes were significantly different from changes induced by two active control stimulation modalities. Closed-loop changes were also specific–beta band power did not change in the brain region used to control the feedback, and alpha power only changed on the array to which we closed the feedback loop. These represent preliminary proof of concept that fast-feedback analog closed-loop stimulation can modify brain oscillations in ways that might be therapeutically useful. They build on other recent demonstrations using alternate forms of feedback to manipulate oscillations in alpha and neighboring frequency regimes [37,40]. Our results also match modeling and empirical studies in which tACS-induced oscillatory enhancements occurred at slightly higher frequencies than the stimulation input [26,57].

The decrease in alpha power from Open-loop and Brain-based stimulation may seem inconsistent with human studies showing alpha power increase from tACS [26,47,48,57] Our protocol, however, was very different from those human experiments. Human open-loop tACS studies usually apply constant stimulation for 20 minutes, following a consensus that this is the maximum safe dose [58]. We applied 10 seconds, a 100-fold smaller dose that has not been clearly effective in human EEG studies [59]. We also report the effect on invasively recorded LFP over millimeter-sized cortical patches, whereas human studies report EEG that reflects much larger-scale synchrony. These two processes involve different physiologic mechanisms. tACS’ effect on LFP has not been well studied, particularly in PFC where the alpha generators are different from visual cortex. The scale of our recordings may also explain why we observed brief after-effects of stimulation while prior human studies reported no effect of 1-second open-loop tACS [28] or of 10-second event-locked closed-loop tACS [59]: we were able to measure more localized effects that may be diluted out before reaching surface EEG. That concept is further supported by our alpha enhancement being limited to Array 1.

Our results do not seem to be explained by changes in overall levels of neuronal firing (spiking). On Array 1, high gamma power (HGP) did not substantially change in any condition, and the conditions did not differ from each other. We did find that Brain stimulation (derived from a previous day’s alpha activity) increased HGP on an array that was not directly under the stimulating electrode, but this occurred even though Array 2 showed weaker alpha effects than Array 1. We may have increased overall activity in some parts of the stimulation zone, but not in an oscillatory fashion. Similarly, the endogenous alpha oscillation did not appear to lock itself to the onset/offset of stimulation. Non-phase-locked power and total power changes were close to each other, while phase-locked alpha power either decreased or remained unchanged during Closed stimulation.

Human tACS experiments often match stimulation to the individual peak alpha frequency. In our study, two of the conditions (Brain and Closed) by definition matched the subject’s individual alpha peak, because they were derived from the macaque’s own alpha oscillation. Open-loop stimulation was a fixed 11.5 Hz frequency that was not tuned to the animal’s physiology. Since Open and Brain both decreased alpha power, and since these conditions did not differ in the Fig 3 analysis, matching tACS to the subject’s alpha peak may be less important for entraining or cancelling small-scale LFP phenomena. Similarly, when our results are examined as spectrograms or time-frequency decompositions, there is no suggestion of narrow-band change or of peak shift. This again might be due to our inherent matching of the endogenous band structure.

This was a limited preliminary study, in which we did not investigate the effect of different stimulation amplitudes (controllable by gain in the filter circuit) or of applying the stimulation for much longer periods. The latter would be an important point to explore and address in future work. We applied very brief stimulation, compared to clinical/human studies that often use 20 minutes of continuous intervention. As a result, we observed small and brief changes in the LFP oscillatory amplitude, on the order of 0.1 to 0.2 dB, and lasting only up to about 1 second post-stimulation. We verified that with longer analysis windows, no changes in LFP power met our pre-determined significance threshold. We would expect longer stimulation bursts to yield much stronger and longer-lasting changes, given that repeated application of tACS can evoke neuroplasticity and that the degree of plasticity appears to be related to the length of tACS bursts [57]. Those larger changes would be necessary for the method to be of value in cognitive neuroscience experiments. If the effect can be made to last for timescales on the order of minutes, pre/post-test designs become feasible. Manipulations that affect alpha and neighboring rhythms have been suggested to improve visual information processing [26], visuo-spatial reasoning [60], and learning [37], and our approach might improve on these effects. In future studies, it would also be valuable to investigate how this broadly distributed stimulation might change functional connectivity within brain networks. Enhancing low-frequency oscillations in a target region might improve that region’s ability to influence downstream targets, but it might equally disconnect it from those targets by enhancing local, autonomous oscillatory generators.

A further limitation of the present approach is that we cannot readily investigate alpha oscillations during the stimulation. Template-subtraction algorithms normally used to remove the effect of tACS are very sensitive to cardiorespiratory artifact [58,61], which we could not readily measure in this preparation. More importantly, two of our three conditions were irregular patterns derived from natural alpha rhythms. Moving-window averaging would not generate a meaningful template for subtraction in this case. Notch filtering would have removed brain signal in the frequency of interest. Future work might address this by changing the method of neurostimulation. A particularly useful approach might be to use a recorded electrical signal to modulate the amplitude of an optogenetic illuminator implanted in the same brain region.

In summary, we showed preliminary evidence that closed-loop feedback through analog filtering can change brain oscillations, with effects that briefly outlast the stimulation period. We also highlighted challenges in moving this technology forward. These include a need to carefully manage phase delays in the processing chain and to measure both local and remote stimulation effects. Given the clinical interest in stimulation customized to endogenous oscillatory frequencies, the optimal circuit implementation would also include rapidly-tunable variable components to match a given patient’s peak. To help achieve that tuning, some components of the system might also be converted back to the digital domain. Recent advances in real-time digital neural signal processing may become fast enough for effective phase-locked stimulation [62]. As we resolve these design issues, this type of rapid feedback stimulation may become a useful tool for cognitive neuroscience and neuropsychiatric treatment.
